# Dirac Spatial Search with Electric Fields

**DOI:** 10.3390/e23111441

**Published:** 2021-10-31

**Authors:** Julien Zylberman, Fabrice Debbasch

**Affiliations:** Sorbonne Université, Observatoire de Paris, Université PSL, CNRS, LERMA, F-75005 Paris, France

**Keywords:** spatial search, quantum walks, Dirac equation, electric field

## Abstract

Electric Dirac quantum walks, which are a discretisation of the Dirac equation for a spinor coupled to an electric field, are revisited in order to perform spatial searches. The Coulomb electric field of a point charge is used as a non local oracle to perform a spatial search on a 2D grid of *N* points. As other quantum walks proposed for spatial search, these walks localise partially on the charge after a finite period of time. However, contrary to other walks, this localisation time scales as $\sqrt{N}$ for small values of *N* and tends asymptotically to a constant for larger *N*s, thus offering a speed-up over conventional methods.

## 1. Introduction

Quantum Walks (QWs) are automata defined on graphs and lattices. These were first considered by Feynman in studying possible discretisations for the Dirac path integral [1,2]. They were later introduced in a systematic manner by Aharonov et al. [3] and Meyers [4], and they have been realized experimentally in a number of ways [5], which include cold atoms [6], photonic systems [7,8] and trapped ions [9,10]. With the recent development of Noisy Intermediate Scale Quantum (NISQ) devices, it is now possible to implement short-depth quantum circuits with several qubits such as QWs [11,12,13,14]. Unitary QWs are also a universal primitive of unitary computation [15,16,17] useful in quantum information and algorithmic development [5,18,19,20,21,22]. Moreover, several spatial search algorithms based on QWs have been proposed [23,24,25,26,27,28,29]. It appears that the most successful strategies consist in using unitary QWs which (i) incorporate the basic idea behind Grover abstract search algorithm [30,31] and (ii) admit, similarly to massless Dirac equation, a linear dispersion relation at large scales [32,33,34]. Some of these unitary QWs work in continuous time [35,36,37,38,39], while others work in discrete time [40,41,42,43,44].

Unitray QWs are also important for quantum simulation [45,46]. In particular, unitary QWs can be used to simulate Dirac fermions interacting with arbitrary Yang-Mills gauge fields [47,48] and arbitrary relativistic gravitational fields [49,50,51,52,53], and steps have been taken to construct alternatives to Lattice Gauge Theories based on Discrete-Time Quantum Walks (DTQWs) [54]. Finally, geometrical aspects of unitary QWs are discussed in [55].

The idea behind the present article is to merge both lines of thought and present a spatial search algorithm based on QWs interacting with a gauge field. In order to keep the discussion as simple as possible, we focus on 2D search on a periodic square grid (2D discrete torus) with *N* points and consider only Discrete-Time Quantum Walks (DTQWs). To permit the introduction of an electric field, the wave function of the walker must have only two components, as the 2D spinors obey the Dirac equation, and not four. The algorithm is based on QWs already introduced in the literature [47]. These walks admit a continuum limit which coincides with the Dirac equation obeyed by a spin 1/2 fermion in flat (1+2)D space-time in the presence of an arbitrary static electric field. This field is encoded in a global phase and acts as the oracle in the search. We first present these DTQWs and recall their continuum limit. We then particularise the electric field to the Coulomb field created by a charge situated at the center of a grid cell Ω and show by numerical simulations that, for spatially homogeneous initial conditions, the algorithm localises partially the walker after a finite time on the four corners of the cell containing the point Ω. The search can then be completed by amplitude amplification. For smaller values of *N*, the partial localisation time of the Dirac walk scales as $\sqrt{N}$, but this tends asymptotically to a constant value independent of *N*. If partial localisation after a finite time is standard for spatial search algorithms based on QWs, the fact that the partial localisation time does not scale as $\sqrt{N}$ for all *N*s but rather tends to a constant for larger *N*s is definitely non standard. All results are finally summed up and discussed with special emphasis on possible extensions.

## 2. Materials and Methods: The Dirac Quantum Walks

### 2.1. Definition

We consider a 2D square grid where the nodes are indexed by the two positive integers $\left(p,q\right)\in N_{M}^{2}$, with *M* an arbitrary strictly positive integer. The integers *p* and *q* can be considered as discrete Cartesian coordinates in 2D space. The total number of points in the grid is $N=M^{2}$, and we impose periodic boundary conditions. Time is also discrete and indexed by j∈N. Given a basis $\left(|b_{L}\rangle ,|b_{R}\rangle \right)$ of an Hilbert space $H_{2}$ named the ‘spin’-space, the wave-function $\psi \in H_{2}$ of the DTQW is represented by its two components $\psi^{L}$ and $\psi^{R}$. The equations of motion of the walks considered in this article are of the form $\psi_{j+1}=U\psi_{j}$ where U is a unitary operator independent of the time *j*. This operator is $U=\exp\left(ieV\right)\cdot R\left(\theta^{+}\right)\cdot S_{q}\cdot R\left(\theta^{-}\right)\cdot S_{p}$ where $S_{p}$ and $S_{q}$ are the standard shift operators defined by the following equations:

(1) $$\begin{array}{ccc} {\left(S_{p}\psi \right)}_{p,q}^{L} & = & \psi_{p+1,q}^{L}\\ {\left(S_{p}\psi \right)}_{p,q}^{R} & = & \psi_{p-1,q}^{L} \end{array}$$

and

(2) $$\begin{array}{ccc} {\left(S_{q}\psi \right)}_{p,q}^{L} & = & \psi_{p,q+1}^{L}\\ {\left(S_{q}\psi \right)}_{p,q}^{R} & = & \psi_{p,q-1}^{L}. \end{array}$$

The operator R(θ) is a rotation in spin space and is represented in the basis $\left(|b_{L}\rangle ,|b_{R}\rangle \right)$ by the matrix

(3) $$R=\left[\begin{array}{cc} \cos(\theta ) & -i\sin(\theta )\\ -i\sin(\theta ) & \cos(\theta ) \end{array}\right]$$

where $\theta^{\pm }=\pm \left(\pi /4\right)-\left(m/2\right)$ with *m* a real positive parameter. The operator *V* is, at each point, proportional to the identity in spin space; thus, ${\left(\exp\left(ieV\right)\psi \right)}_{p,q}=\exp\left(ieV_{p,q}\right)\psi_{p,q}$ where *e* is another arbitrary real parameter. The interpretation of *m* and *e* will be made clear below.

### 2.2. Continuum Limit

The continuum limit describes situations where the wave-function ψ of the walk varies on time-scales and spatial scales much larger than the step of the grid. Suppose that there exists a real positive number ϵ much lower than unity such that, in a certain domain in (j,p,q)-space, the wave-function ψ varies on scales of order $\epsilon^{-1}$ in its three variables *j*, *p* and *q*. Then, define the ‘slow’ variables t=ϵj, x=ϵp and y=ϵq so that ψ varies on scales of order unity in these new variables. Suppose also that, in the same domain, the quantities eV and *m* are of order ϵ and write $eV=\epsilon e\tilde{V}$ and $m=\epsilon \tilde{m}$. The discrete equations of the motion for the walk can then be expanded in powers of ϵ around ϵ=0. This expansion delivers the identity ψ=ψ at zeroth order in ϵ but, at first order, one obtains he following.

(4) $$\begin{array}{ccc} \left(\partial_{t}-ie\tilde{V}\right)\psi^{L}-\partial_{x}\psi^{L}-i\partial_{y}\psi^{R}+i\tilde{m}\psi^{R} & = & 0\\ \left(\partial_{t}-ie\tilde{V}\right)\psi^{R}+\partial_{x}\psi^{R}+i\partial_{y}\psi^{L}+i\tilde{m}\psi^{L} & = & 0. \end{array}$$

This equation coincides with the flat (1+2)D space-time Dirac equation for a spinor of mass $\tilde{m}$ and electric charge −e propagating in the electric potential $\tilde{V}$. More details on the calculations of the continuous limit can be found at [50,56].

## 3. Results: Search with Coulomb Potential

We now particularise the discussion to the following choice:

(5) $$V_{p,q}=Q{\left({\left(p-\Omega_{p}\right)}^{2}+{\left(q-\Omega_{q}\right)}^{2}\right)}^{-1/2},$$

except on the borders of the grid, where we impose the potential to vanish identically, thus preserving periodicity in both *p* and *q*. The above expression coincides with the Coulomb potential created by a point charge *Q* situated at point $\Omega =(\Omega_{p},\Omega_{q})$. To ensure that the walk is defined at all points in the grid, the point Ω where *V* diverges must not be on the grid. A simple possibility is to choose Ω at the center of a grid-cell equidistant from the four vertices of this cell. The distance in the (p,q) Euclidean plane between Ω and these 4 points is then $1/\sqrt{2}$, and the maximum value taken by the function *V* on the grid is the following.

(6) $$V_{max}=Q\sqrt{2}.$$

At given Ω, *m* and *N*, the walk is entirely controlled by the initial condition and the parameter a=eQ. The maximum value of the global phase is $\alpha_{max}=eV_{max}=eQ\sqrt{2}$.

As is usual in spatial search problems, we choose initial conditions that are uniform on the grid and in spin state [30,31]. Considering the chosen potential, the algorithm can be considered successful if it localises the walker on the four vertices of the cell centred on Ω. Let $P_{j}^{*}$ be the probability of finding the walker at time *j* on any of these four vertices. The explicit expression of $P_{j}^{*}$ is:

(7) $$P_{j}^{*}=P_{j,\Omega_{p}+1/2,\Omega_{q}+1/2}+P_{j,\Omega_{p}-1/2,\Omega_{q}+1/2}+P_{j,\Omega_{p}+1/2,\Omega_{q}-1/2}+P_{j,\Omega_{p}-1/2,\Omega_{q}-1/2}$$

with

(8) $$P_{j,p,q}=|\psi_{j,p,q}^{L}{|}^{2}+|\psi_{j,p,q}^{R}{|}^{2}.$$

The probability $P_{j}^{*}$ depends in a non trivial manner on the free parameters Ω, *m*, *N* and eQ. Exploring systematically this parameter space is out of the scope of this article. What follows is a synthetic presentation of some features observed in extensive numerical simulations.

**Figure 1:**
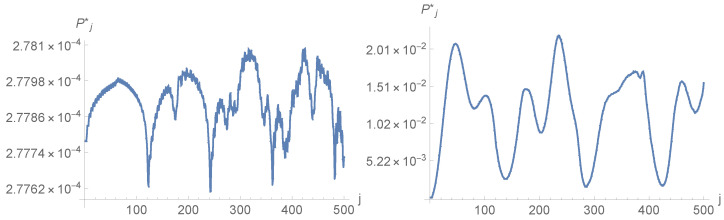
Evolution of $P_{j}^{*}$ with time *j* for $N=120^{2}$, $\Omega =\Omega_{0}$, m=0, eQ=0.01 (**left**) and eQ=1 (**right**).

**Figure 2:**
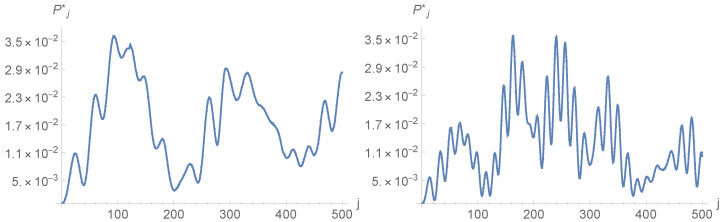
Evolutions of $P_{j}^{*}$ with time *j* for $N=120^{2}$, $\Omega =\Omega_{0}$, eQ=1 and mass m=0.08 (**left**) or m=0.25 (**right**).

As expected from the behaviour of other QW based search algorithms, at fixed values of the parameters Ω, *m*, *N* and a=eQ, the probability $P_{j}^{*}$ displays strong oscillations in *j*. For example, Figure 1 displays two typical evolutions of $P_{j}^{*}$ with *j*, both obtained on a grid of $N=120^{2}$ points, with m=0. and with $\Omega =\Omega_{0}$ located at the center of the central grid cell but for different values of *a*. Changing Ω does not change $P_{j}^{*}$ too much, at least if one does not proceed too close to the border of the grid (data not shown). However, changing *m* typically increases the oscillation frequency of $P_{j}^{*}$, as exemplified Figure 2, which plots $P_{j}^{*}$ against time *j* for $N=120^{2}$, $\Omega =\Omega_{0}$, m=0.08 and m=0.25. The introduction of a non-vanishing mass provides greater inertia to the walker, slowing down the localisation process. In Figure 2, the value ∼0.21 is reached by function $P_{j}^{*}$ at a later time when compared to the right curve of Figure 1. Note also that the values reached by $P_{j}^{*}$ are larger when the mass vanishes.

**Figure 3:**
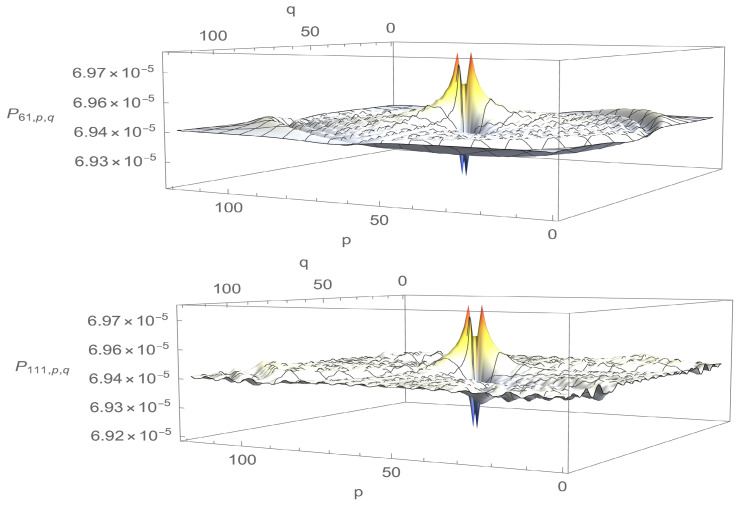
Density profiles at time j=61 corresponding to the first maximum of $P_{j}^{*}$ (**up**) and at time j=111 corresponding to the first minimum of $P_{j}^{*}$ (**down**) for eQ=0.01, $N=120^{2}$ and $\Omega =\Omega_{0}$.

**Figure 4:**
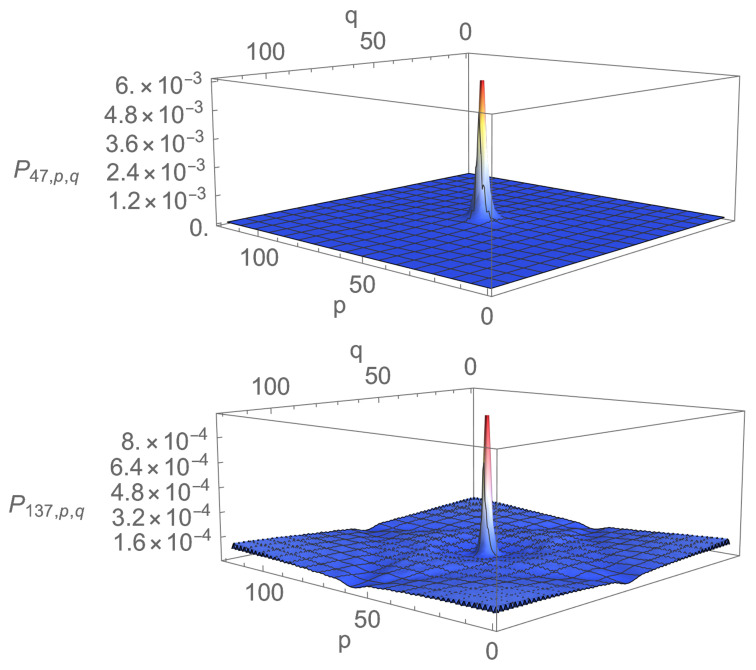
Density profiles at time j=47 corresponding to the first maximum of $P_{j}^{*}$ (**up**) and time j=137 corresponding to the first minimum of $P_{j}^{*}$ (**down**) for eQ=1, $N=120^{2}$ and $\Omega =\Omega_{0}$.

**Figure 5:**
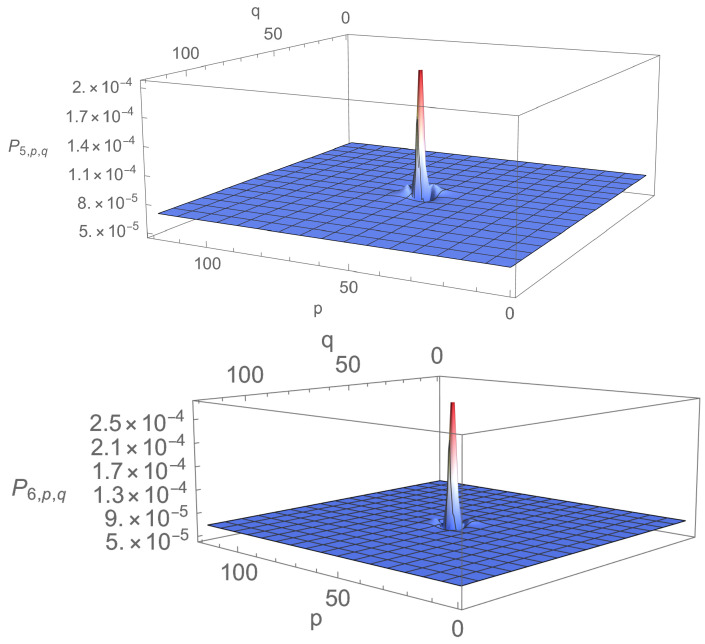
Density profiles at times j=5 (**up**) and j=6 (**down**) for eQ=1, $N=120^{2}$ and $\Omega =\Omega_{0}$.

Density profiles corresponding to the first maximum and the first minimum of $P_{j}^{*}$ are presented in Figure 3 and Figure 4. It is obvious from the figures that localisation is much more efficient for a=1 than for a=0.01. In particular, for a=0.01, localisation does occur on two of the four vertices, but it is accompanied by rather strong anti-localisation on the other two and the background probability, i.e., the probability of finding the walker elsewhere than around $\Omega_{0}$ is not negligible. Furthermore, there is not much difference between the density profiles corresponding to the first maximum and the first minimum of $P_{j}^{*}$: Peaks have more or less the same height, and density displays ripples and bumps outside the peaks. The picture changes drastically for a=1. There is now practically no anti-localisation, the peak at time j=47 when $P_{j}^{*}$ is maximal is approximately 10 times higher than the peak at time j=137 when $P_{j}^{*}$ is minimal, and the density outside the peaks is nearly flat and practically vanishes, even when $P^{*}$ is minimum. In this case, localisation actually happens well before $P^{*}$ reaches its first maximum (see Figure 5); once installed, localisation remains at all explored times.

**Figure 6:**
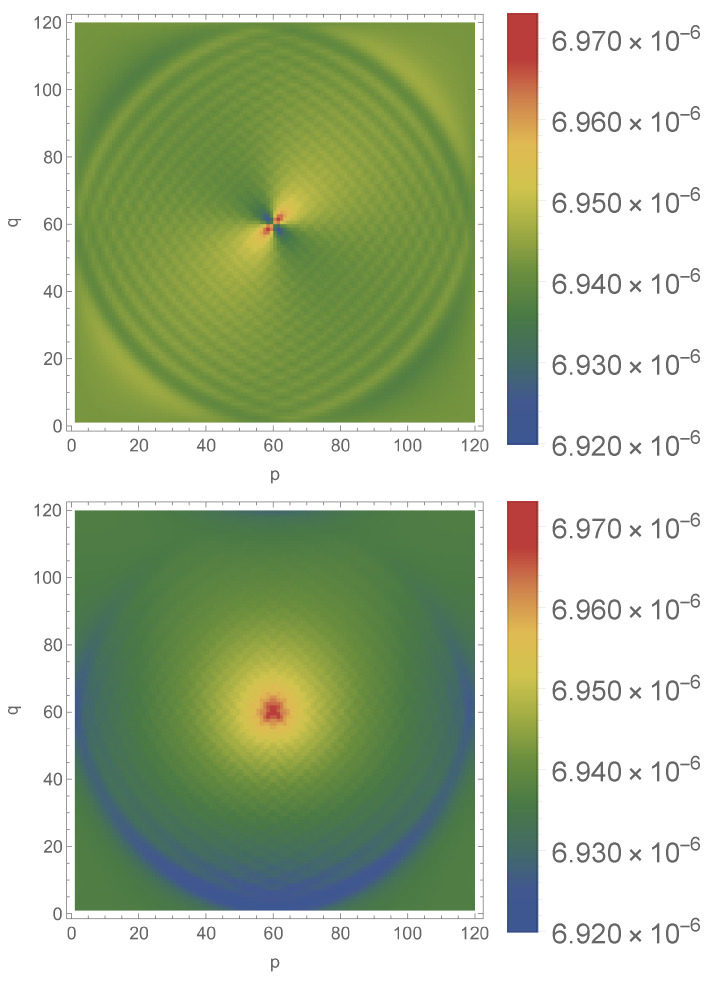
Density contours at time j=61 corresponding to the first maximum of $P_{j}^{*}$ for eQ=0.01, $N=120^{2}$, $\Omega =\Omega_{0}$ and two different initial conditions: $\psi^{L}=\psi^{R}$ (**up**) and $\psi^{R}=0$ (**down**).

The density profiles displayed in Figure 3, Figure 4 and Figure 5 are non symmetric around the central point Ω; in particular, the walker does not distribute evenly among the four vertices that surround Ω. This is due to the choice of initial condition in spin state. The upper part of Figure 6 offers a contour version of the upper Figure 3 and corresponds to an initial condition symmetric in $\psi^{L}$ and $\psi^{R}$. Switching to an initial condition with vanishing $\psi^{R}$ does not change the time-evolution of $P^{*}$ (data not shown) but produces the other contour plot in Figure 6 where the central peak is nearly symmetric.

A final comment on the density profiles is in order. The absolute values taken by the density $P_{j,p,q}$ may appear to be rather small. The main reason for that is the total number of points $N=120^{2}$ over which the walker moves. A uniform probability spread uniformly over $120^{2}$ points amounts to $6.94\times 10^{-5}$. The density profiles corresponding to a=0.01 reveal that the peaks are then approximately 4.3% above this value. However, the peak for a=1 at time t=47 corresponding to the first maximum of $P_{j}^{*}$ is approximately $0.006∼86\times 6.94\times 10^{-5}$, corresponding to an increase of 8500% with respect to uniform spreading, which is quite substantial. Whatever the increase, the absolute value of 0.006 may still be considered small compared to unity. However, it is not vanishingly small. Moreover, as already indicated in the introduction and as discussed in the final section, the search procedure offered by the DTQWs presented cannot be considered complete, since the probability of finding the walker on the four vertices surrounding Ω is never equal to unity. The search should, therefore, be complemented by amplitude amplification.

**Figure 7:**
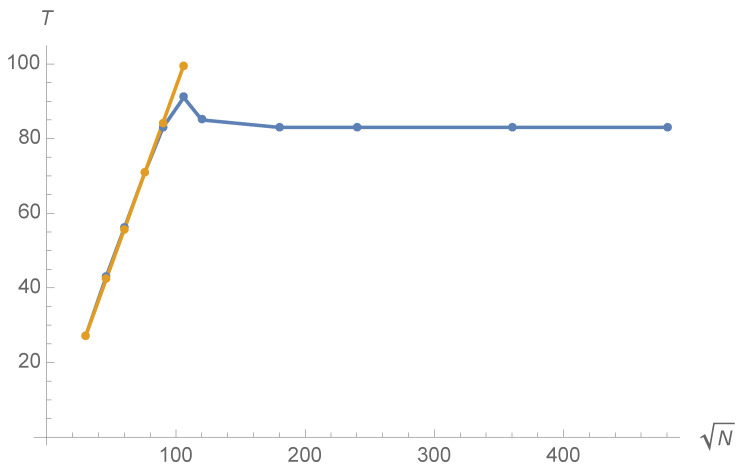
Time *T* for the first maximum of ${\bar{P}}_{j}^{*}$ in function of $\sqrt{N}$ for eQ=0.9, $\Omega =\Omega_{0}$ and m=0. The function fitting *T* for small *N* is approximately $0.96\sqrt{N}-1.66$ and appears in yellow.

**Figure 8:**
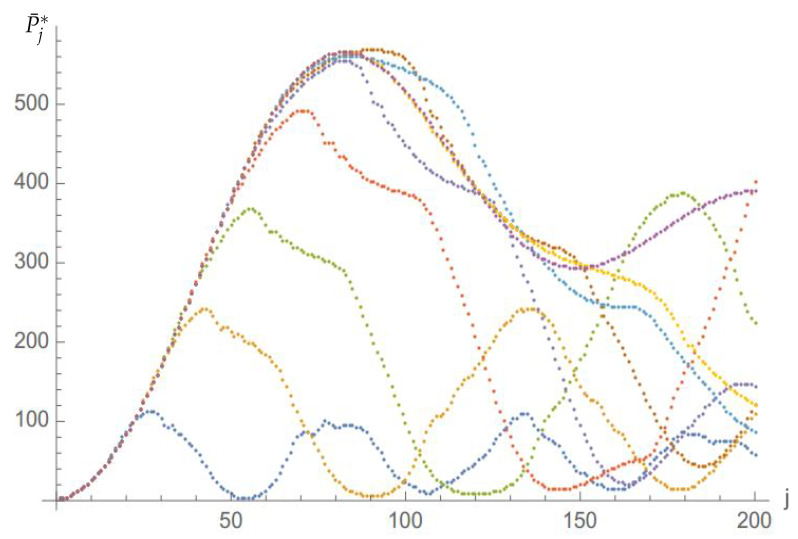
Renormalised probability ${\bar{P}}_{j}^{*}$ against time *j* for eQ=0.9, $\Omega =\Omega_{0}$ and $N=30^{2}$ (blue), $N=46^{2}$ (orange), $N=60^{2}$ (green) and $N=76^{2}$ (red), $N=90^{2}$ (navy blue), $N=106^{2}$ (brown), $N=120^{2}$ (cyan), $N=180^{2}$ (yellow) and $N=240^{2}$ (purple).

Let us now explore how the time *T* at which $P_{j}^{*}$ reaches its first maximum evolves with *N*. Typical results are displayed in Figure 7 and Figure 8 for $\Omega =\Omega_{0}$ and a=0.9. Figure 7 displays the time *T* of the first maximum of $P_{j}^{*}$ as a function of *N*. At small *N*, *T* increases as $\sqrt{N}$ but *T* is asymptotically constant. This unexpected behaviour has been observed numerically for all values of *a*; the greater *a* is, the sooner the asymptotic regime in which *T* is independent of *N* is reached. For example, for a=1, the asymptotic regime is reached around N=30.

Figure 8 displays the renormalised probability ${\bar{P}}_{j}^{*}=4P_{j}^{*}/N$ plotted against time for $N=30^{2}$ (blue), $N=46^{2}$ (orange), $N=60^{2}$ (green), $N=76^{2}$ (red), $N=90^{2}$ (navy blue), $N=106^{2}$ (brown), $N=120^{2}$ (cyan), $N=180^{2}$ (yellow) and $N=240^{2}$ (purple). The $\sqrt{N}$ scaling and the constant asymptotic behaviour can naturally be observed in this figure too. Figure 8 also shows that, quite remarkably, the function ${\bar{P}}^{*}$ is essentially independent of *N* on the left of the first maximum. Note also that the short *N*-scaling does *not* involve logN.

Let us end this section by a qualitative comment for explaining how the localisation time becomes independant of *N* due to the finite velocity of the walk. Fix all values of the parameters except *N* and suppose that, for some value $N_{0}$ of N, the first maximum of $P_{j}^{*}$ is reached at time $T_{0}$. Since DTQW is a discrete version of the relativistic Dirac equation, it essentially propagates at a finite velocity which, in the units used in this article, is equal to unity. Thus, in time $T_{0}$, the peak around Ω has only been influenced by points at distance $∼T_{0}$. If $\sqrt{N_{0}}/2$ is sufficiently lower than $T_{0}$, increasing *N* will allow points around Ω to observe more distant points and will presumably modify $T_{0}$. However, suppose $\sqrt{N_{0}}/2$ is larger than $T_{0}$. Then, increasing *N* will not modify the dynamics of the peak until times later than $T_{0}$; thus, $T_{0}$ will be independent of *N* for *N* larger than $N_{0}$. Observe now Figure 7. The time *T* becomes independent of *N* around $N=160^{2}$ and is then approximately equal to $T_{a}∼84∼160/2$, which seems to confirm the above reasoning. If this line of reasoning is correct, any walk that propagates at finite velocity *c* and for which one can find an $N_{0}$ such that $\sqrt{N_{0}}/2>cT_{0}$ will present the same asymptotic property. Intuitively, the existence of such an *N* seems linked to the rapid convergence towards Ω exemplified in Figure 5.

## 4. Discussion

Let us now discuss these results and mention some natural extensions. One should first stress that the DTQWs considered in this article, as previous QWs used in spatial search algorithms, never fully localise on the desired points. In the present context, localisation means that the probability of finding the walker on the desired point is substantially higher than the background probability of finding it at any other point. Fully localising the walk would require adding a step commonly called amplitude amplification.

The most interesting aspect of these walks is their behaviour for large enough values of *N*. Indeed, localisation times for other spatial search algorithms scale as $\sqrt{N}$ or $\sqrt{N}\log N$. The partial localisation time of these new walks does scale as $\sqrt{N}$ for small enough *N* but appears to saturate a constant value for larger *N*. This is possibly the most remarkable property of these walks. Of course, this does not contradict the theorem that states that an optimal quantum search takes $O(\sqrt{N})$ steps because the localisation we are speaking of is not total, and completing it with amplitude amplification would take $O(\sqrt{N})$ steps. Thus, the saturation property of the walks introduced in this article does not change the order of magnitude of a the minimal time necessary to perform a full quantum search. However, for large enough values of *N*, it makes the first step of the spatial search much faster than expected and realised in other quantum walks. Stopping after this step may even be enough for some applications.

The results presented in this article should first be generalised not only to higher dimensions but also to general lattices or graphs and and more varied initial conditions, especially in spin space. The simulations presented in this article show that it is possible to use artificial gauge fields as oracles to perform efficient spatial searches. One could also consider using other artificial gauge fields, including magnetic fields, general Yang-Mills fields and gravitational fields, as oracles in quantum search problems. Moreover, one wonders whether problems more complex than the one studied in the present, such as for example the motion of several particles in a gauge field they self-consistently generate, are also susceptible of quantum algorithmic interpretation. As for now, this article adds to the literature showing that physics can be a source of inspiration to develop new strategies in quantum information. Other examples include applications of quantum tunelling [57] and of Anderson localization [58,59].

## Data Availability

Data are available from the authors upon request.

## Abbreviations

The following abbreviations were used in this manuscript:

QW	Quantum Walk;
DTQW	Discrete Time Quantum Walk.
